# Investigating the Added Value of the EQ-5D-5L With Two Bolt-On Items in Patients With Hemophilia

**DOI:** 10.3389/fmed.2021.707998

**Published:** 2021-08-05

**Authors:** Richard Huan Xu, Dong Dong, Nan Luo, Renchi Yang, Junshuai Liu, Shuyang Zhang

**Affiliations:** ^1^Department of Rehabilitation Sciences, The Hong Kong Polytechnic University, Kowloon, Hong Kong; ^2^JC School of Public Health and Primary Care, The Chinese University of Hong Kong, Shatin, Hong Kong; ^3^Shenzhen Research Institute, The Chinese University of Hong Kong, Shenzhen, Guangdong, China; ^4^Saw Swee Hock School of Public Health, National University of Singapore, Singapore, Singapore; ^5^Thrombosis and Hemostasis Center, Institute of Hematology and Blood Diseases Hospital, Chinese Academy of Medical Sciences and Peking Union Medical College, Tianjin, China; ^6^Beijing Society of Rare Disease Clinical Care and Accessibility, Beijing, China; ^7^Department of Cardiology, Peking Union Medical College Hospital, Peking Union Medical College and Chinese Academy of Medical Science, Beijing, China

**Keywords:** EQ-5D, health-related quality of life, bolt-on items, psychometric properties, hemophilia

## Abstract

**Objective:** This study examined the impact of adding two condition-specific bolt-on items to the EQ-5D-5L and assessed their psychometric properties in patients with hemophilia.

**Methods:** The data were obtained from a nationwide cross-sectional online survey of patients with hemophilia in China. Self-reported and proxy-reported data were analyzed separately. Ceiling effect, informativity, and discriminatory power of the EQ-5D-5L with two bolt-on items, dignity (DG), and bleeding (BL), were examined. Spearman's rank correlation (rho) was used to assess the associations of the EQ-5D-5L and two bolt-on items with the Hemophilia Quality of Life Questionnaire for Adults (Haem-A-QoL) and SF-12. Multiple regression analysis was performed to evaluate the explained variance of the EQ-5D-5L and bolt-on items in predicting EQ-VAS scores.

**Results:** A total of 895 patients and 222 caregivers completed the questionnaire. The ceiling effect decreased from 1.9 to 0.6% and 5.9 to 0.9% when using the EQ-5D-5L and the EQ-5D-5L with two bolt-on items among participants with both self- and proxy-completed questionnaires. Both DG and BL were strongly correlated with Haem-A-QoL sum score [rho: DG = 0.64 (patient) vs. 0.66(proxy); BL = 0.49 (patient) vs. 0.31 (proxy)], SF-12 mental component [rho: DG = −0.36 (patient) vs. −0.41 (proxy); BL = −0.53 (patient) vs. −0.57(proxy)], and SF-12 physical component [rho: DG = −0.61 (patient) vs. −0.61 (proxy); BL = −0.35 (patient) vs. −0.39 (proxy)]. Known-group comparisons confirmed that the two bolt-on items had satisfactory discriminatory power. Multiple regression analysis indicated that adding two bolt-on items significantly increased the ability to predict EQ-VAS scores. The adjusted *R*^2^ increased by 8.2 and 8.8% for reports completed by the patients or patients' proxy respondents, respectively.

**Conclusion:** Adding the DG and BL bolt-on items can increase performance on the EQ-5D-5L in patients with hemophilia. A future valuation study will be carried out.

## Introduction

Hemophilia, which has two main types (A and B), is a rare hematological disease that primarily affects males (1). Globally, hemophilia affects approximately 400,000 people, with an estimated prevalence of 1 in 5,000 male live births for type A and 1 in 30,000 live births for type B (2). Hemophilia undoubtedly has a negative impact on patients' health-related quality of life (HRQoL) and psychological well-being due to its chronicity, symptoms, and complications (3). Life expectancy in hemophilia varies, depending on whether patients receive appropriate treatment. Overall, the mortality rate of male patients is about twice the rate of healthy men; whereas for those with severe hemophilia, the rate could be four to six times higher (4). In addition, comorbidity with other diseases is also regarded as a potential but uncertain factor that may affect the mortality rate of patients with hemophilia as well.

Bleeding episodes produced by abnormal clotting factors are hallmarks of hemophilia. The severity of hemophilia varies, from bleeding after surgery to spontaneous bleeding, and depends on the level of clotting factors (5). It is usually internal and causes joint and muscle damage and pain (1). In some cases, repeated bleeding in the same body part can cause chronic inflammation and reduce joint flexibility and muscle mobility, causing permanent disability (6, 7). Previous studies have shown that bleeding disorders lead to a significantly poor physical HRQoL, and alleviating these symptoms is highly likely to improve patients' physical HRQoL and well-being (8–10).

Survival rates for patients with hemophilia have increased significantly (11); however, longer life expectancy has increased the risk of developing some chronic psychiatric, psychological, and social support problems, all of which can lead to loss of dignity (12, 13). One previous study indicated that a large number of patients with hemophilia are disabled to some extent due to the complications from treatment. As a result, these patients cannot live in accordance with their standards and values, and the loss of self-esteem jeopardizes their HRQoL and well-being (14). Ganzini et al. also found that loss of dignity is one of the main reasons that the family of hemophiliacs have advocated for legalized physician-assisted death (15, 16). Further studies have demonstrated that low dignity is strongly associated with poor HRQoL (17, 18). However, few clinical or social intervention studies have been carried out on the topic.

The EQ-5D, one of the most widely used generic preference-based measures (PBMs) worldwide (19–22), is increasingly used to measure HRQoL in different patient groups (23, 24). It can generate a summarized utility score that aids decision-makers in allocating scarce healthcare resources (25). Unlike condition-specific PBMs, such as the Hemophilia Quality of Life Questionnaire for Adults (Haem-A-QoL), generic PBMs are intended for use across conditions and treatments and to provide consistency and comparability for economic evaluations (26). Although the use of the EQ-5D in hemophilia is growing, there are notable gaps.

The EQ-5D was designed for both simplicity and comprehensiveness in the measurement of HRQoL (27). To ensure simplicity, the descriptive system comprises only five items to reflect different dimensions of HRQoL, four of which measure the physical aspect of HRQoL and one of which measures the mental aspect of HRQoL. For comprehensiveness, the EQ-5D tries to cover all the dimensions of health in general, rather than specific aspects. Hence, it can provide consistency and comparability within and between different populations and settings. However, a growing number of studies have indicated that the EQ-5D lacks sensitivity and appropriateness for measuring changes in HRQoL in some specific patient groups (26, 28–30). To increase the ability of the EQ-5D to capture the important variations of HRQoL in patients with specific conditions, adding condition-specific bolt-on items were introduced. The EQ-5D with the two bolt-on items has been shown to provide valid and reliable results that may increase the sensitivity of the EQ-5D to capture the condition-specific changes in HRQoL in a specific patient group (31–33). Using the bolt-on method, the integral structure of the EQ-5D descriptive system can be maintained, and simultaneously, the predictive ability to estimate the change in HRQoL in a subpopulation is improved.

For patients with hemophilia, the EQ-5D may not be able to detect certain changes in HRQoL due to symptoms unique to hemophilia or side effects resulted from its treatment. Using the bolt-on approach can ensure comparability, transparency, and consistency when measuring HRQoL across different hemophilia-related interventions. Previous studies indicated that adding condition-specific bolt-on items to the EQ-5D may undermine the cross-program comparability because different interventions measure different dimensions of health (34, 35), and a new algorithm including the bolt-on items is therefore needed to calculate utility scores to support cost-utility analysis. However, when performing economic evaluations, the so-called orphan drugs and products for rare diseases (RDs) including hemophilia are often found not being cost-effective if measured under standard thresholds (36). Hence, without disease-specific bolt-on items, the risks of not responding to the patients' needs will be increased and equal access to medical care will be hindered (37).

To develop an independent value set that considers specific symptoms of hemophilia and side-effects caused by its treatment, which is vital to assess the cost-effectiveness of different hemophilia-related interventions, it is important firstly to confirm the validity of the additional dimensions added to the EQ-5D so that a local value set can be developed based on this expanded instrument. In this study, two hemophilia-specific bolt-on items were added to the EQ-5D: bleeding (BL) and dignity (DG). They were identified and developed through a literature review, focus group interviews, and expert discussions aimed at specifically measuring the changes in physical and mental HRQoL for hemophiliacs that might be insufficiently captured by the original EQ-5D. Thus, the aims of this exploratory study were to examine the impact of adding two bolt-on items to the EQ-5D and to assess the psychometric properties of the EQ-5D with bolt-on items in a sample of Chinese patients with hemophilia.

## Methods

### Research Population and Data Collection

The data used in this study were obtained from an online nationwide cross-sectional survey to investigate the health and socioeconomic status of patients living with hemophilia in China. The survey was conducted between August 2019 and December 2019. Research team had collaborated with the “*Home of Hemophilia,”* the biggest hemophilia patient organization in China, to perform data collection. All the participants were recruited via the patient organization's internal network. A recruitment advertisement was sent to its registered members via its internal member management platform. Inclusion criteria were: (1) ≥18 years; (2) no cognitive problems (screened by the patient organization and self-reported by the patients); (3) able to provide informed consent. Interested and eligible members (assessed by the patient organization) were invited to join in an online “surveying group” and a link of questionnaire was provided in that group. Information on patients' demographics, socioeconomic status, diagnosis, and treatment status, HRQoL, and access to and use of healthcare services were collected. Survey procedure, implementation, and quality control were defined and monitored by the survey committee, which was composed of medical specialists dealing with hemophilia, leaders of patient organizations, and our research team. The first page of the online questionnaire was the consent form, and all the participants were forced to read through it. The survey would not begin until participants clicked the “Agree” button at the end of that page. They were also provided an option on “Do not agree and leave.” Since some patients might not be able to complete the questionnaire all by themselves due to poor health status, their main caregivers would be recruited to complete the survey for them. At the beginning of the survey, participants were therefore required to indicate their identity as a patient or a caregiver. Then patients and caregivers were asked to complete different versions of the questionnaire, and their responses were coded as either self- or proxy-completed data. The Institutional Review Board Ethics Committee of the Chinese University of Hong Kong and Peking Union Medical College Hospital approved the study protocol (Ref no.: SBRE-18-268 and SK814). This study was carried out in accordance with the 1964 Declaration of Helsinki and its later amendments. Written informed consent was provided by all participants.

### HRQoL Measurement

#### EQ-5D-5L

In this study, we used the five-level version of the EQ-5D (EQ-5D-5L). It has two sections: a descriptive system section and a visual analog scale (EQ-VAS). The descriptive system section consisted of five items, one on each of the following: mobility (MO), self-care (SC), usual activities (UA), pain/discomfort (PD), and anxiety/depression (AD). For each item, responses are based on a 5-point scale, ranging from “no problem” (1) to “extreme problems/” (5). A profile of “11111” indicates that the patient has no problems in all five items and is the best possible health state. The EQ-VAS reflects a person's overall health on a vertical visual analog scale, ranging from 0 to 100, with a higher score indicating a better imagined health state (38).

#### Bolt-On Items to the EQ-5D-5L

Two condition-specific bolt-on items to the EQ-5D-5L, the DG and BL, were developed based on a sophisticated process including literature review, patient focus group interview, and expert discussion. Two bolt-on items were designed to reflect general changes in psychosocial and physical HRQoL in hemophiliacs, respectively. Dignity is a complicated concept that comprises a number of related issues, such as self-respect, self-concept, self-confidence, and self-esteem (39–42). Given that patients with RDs, including hemophilia, have to deal with dignity-related issues both during doctor visits and daily life, we decided to follow the Dixon et al.'s work (42) and developed this bolt-on item to directly ask patients about their experiences and feelings about DG in general. Bleeding was defined as patients experiencing all types of bleeding, including bleeding into the joints, skin, and mouth; bleeding of the mouth and gums; bleeding after circumcision; bleeding after receiving shots; blood in the urine or stool; and hard-to-stop nosebleeds (43). Patients were asked to indicate how severe the problem of bleeding they experience in daily life on the day of survey, regardless the type or source of bleeding or control effectiveness. Both the DG and BL items were framed the same as the other items of the EQ-5D-5L, with the same number of response options. Responses to the DG item was worded as follows (Chinese version was presented to all participants): I live with full dignity,” “I live with many dignities,” “I live with some dignities,” “I live with few dignities,” and “I live with no dignity.” Responses to the BL item were as follows: “I have no bleeding problems,” “I have slight bleeding problems,” “I have moderate bleeding problems,” “I have severe bleeding problems,” and “I have extreme bleeding problems.”

#### Haem-A-QoL

The Haem-A-QoL is a commonly used instrument to assess the HRQoL for adult patients with hemophilia (44–47). It consists of 46 items grouped into 10 dimensions. The scale specifically assesses HRQoL of patients with hemophilia (48). These include physical health (PHYS, 5 items), feelings (FEEL, 4), view of self (VIEW, 5), sports and leisure (SPORT, 5), work and school (WORK, 4), dealing with hemophilia (DEAL, 3), treatment (TREAT, 8), future (FUTURE, 5), family planning (FAMPL, 4), and partnership and sexuality (SEXUAL, 3). The sum score of the Haem-A-QoL ranges from 0 to 100 and is obtained by summing up the scores of all 10 subscales, with a higher score indicating poorer HRQoL.

#### SF-12

The SF-12 is one of the most widely used generic non-preference based measure, which has been used to assess HRQoL in patients with hemophilia and proved having satisfactory performance (49, 50). It consists of 12 questions on 8 dimensions of physical and mental health: general health (GH), physical functioning (PF), role physical (RP), and body pain (BP), vitality (VT), social functioning (SF), role emotional (RE), and mental health (MH). Scores are reported as physical and mental component summary scores (PCS and MCS) (51).

### Statistical Analysis

Self-completed and proxy-completed data were analyzed separately. Descriptive analysis was used to describe the participants' characteristics. The proportion of participants' responses on each level of the EQ-5D-5L and bolt-on items are presented as percentages. We calculated the proportion of participants with the best health state, as measured with the EQ-5D-5L and EQ-5D-5L with two bolt-on items. For the EQ-5D-5L, the best health state was indicated by a profile of “11111.” For the EQ-5D+DG and EQ-5D+BL, it was indicated by a profile of “111111.” For the EQ-5D+DG+BL, it was indicated by a profile of “1111111.”

Convergent validity was determined by examining the correlations between the EQ-5D-5L with two bolt-on items and the Haem-A-QoL and SF-12. Spearman rank correlation (*r*s) was used to confirm the strength of the associations, where 0.25 < *rs* < 0.5, and *rs* ≥ 0.5 were identified as moderate and strong correlations, respectively (52). We assumed that the EQ-5D-5L with bolt-on items would positively correlate with the Haem-A-QoL sum score, but negatively correlate with PCS and MCS of the SF-12. We further posited that the DG item would show a stronger association with MCS than PCS, and that the BL item would show a stronger association with PCS than MCS. Known-group validity was assessed by testing the priori hypotheses that patients with poorer health status had more problems with dignity and bleeding. A chi-squared test was used to assess the discriminatory ability of the DG and BL items to differentiate patients known to differ in terms of severity of hemophilia, disabling levels, and comorbidity.

We performed both univariable and multivariable linear regression analysis to compare the exploratory power of the EQ-5D-5L and EQ-5D-5L with two bolt-on items. The EQ-VAS was used as the dependent variable in all the models. For univariable analysis, five items of the EQ-5D-5L and another two bolt-on items were analyzed separately (seven models). For multivariable analysis, another five models were developed. (a) DG+BL model, (b) EQ-5D model (MO+SC+UA+PD+AD), (c) EQ-5D+DG model, (d) EQ-5D+BL model, and (e) full model (MO+SC+UA+PD+AD+DG+BL). *R*-squared (*R*^2^) and adjusted *R*-squared (adjusted *R*^2^) were used to determine the exploratory ability of the models. The Shannon index (*H*′) and the Shannon evenness index (*J*′) were used to assess the classification efficiency of the EQ-5D-5L and EQ-5D-5L with two bolt-on items, respectively. They provided information to assess the ability of the measurements to gauge the diversity of patients (53). All analyses were performed using R software (R Foundation, Vienna, Austria). Statistical significance was set at *p* ≤ 0.05.

## Results

### Participant Characteristics

Data from 895 patients and 222 caregivers who completed the survey were included in our analysis. Approximately 85.8% of self-completed questionnaires and 85.4% of proxy-completed questionnaires were from type A patients. Approximately 72.1% of patients who self-completed their questionnaire were aged between 21 and 40 years, and 46.9% completed secondary or above education. More than 60% were rural residents, and nearly 70% reported a family income of <50,000 CNY ($7,800 USD) per year. For participants whose questionnaires had been completed by proxy, more than 70% were aged under 30 years, and 53.6% were rural residents (Table 1).

**Table 1:** Respondents' characteristics.

	**Overall respondents**	**Self-completed respondents**		**Proxy-completed respondents**		***p*-value**
		***n***	**%**	***n***	**%**	
**Sex**						
Male	1,117	895	100	222	100	–
**Ethnic group**						
Han	1,064 (95.3)	853	95.3	211	95.0	0.87
Others	53 (4.7)	42	4.7	11	5.0	
**Age**						
≤20	164 (14.7)	72	8.0	92	41.4	<0.001
21–30	427 (38.2)	362	40.4	65	29.3	
31–40	324 (29)	287	32.1	37	16.7	
41–50	141 (12.6)	125	14.0	16	7.2	
≥51	61 (5.5)	49	5.5	12	5.4	
**Education**						
No/Primary	581 (52)	475	53.1	106	47.7	0.06
Secondary	399 (35.7)	305	34.1	94	42.3	
Tertiary or above	137 (12.3)	115	12.8	22	9.9	
**Family register**						
Urban resident	451 (40.4)	348	38.9	103	46.4	0.05
Rural resident	666 (59.6)	547	61.1	119	53.6	
**Family income**						
≤10,000	183 (17)	151	17.5	32	14.7	0.9
10,001–30,000	328 (30.4)	260	30.2	68	31.3	
30,001–50,000	257 (23.8)	203	23.6	54	24.9	
50,001–80,000	120 (11.1)	94	10.9	26	12.0	
80,001–100,000	98 (9.1)	78	9.1	20	9.2	
≥10,0001	92 (8.5)	75	8.7	17	7.8	
**Type**						
Type A	946 (85.8)	759	85.8	187	85.4	0.43
Type B	150 (13.6)	122	13.8	28	12.8	
Others/uncertain	4 (0.4)	2	0.2	2	0.9	

*Family income per year (Chinese Yuan)*.

### Frequency of Health States and Ceiling Effects

Table 2 demonstrates that among patients who self-completed their questionnaire, only 15% and 2% responded that they had no problems for the DG and BL items, respectively, which indicated an acceptable ceiling effect. For the EQ-5D-5L, 1.9% of patients reported a perfect health state (i.e., “11111”); however, the proportion was lower, at 0.6%, for the EQ-5D+DG+BL (i.e., “1111111”). For patients whose questionnaire had been completed by proxy, a total of 5.9% of participants reported a perfect health state on the EQ-5D-5L, whereas the proportion was lower, at 0.9%, for the EQ-5D+DG+BL.

**Table 2:** Proportion of EQ-5D-5L items and the two bolt-on items.

	**%**						
	**EQ-5D-5L descriptive system**					**EQ-5D-5L bolt-on items**	
	**MO**	**SC**	**UA**	**PD**	**AD**	**DG**	**BL**
**SELF-COMPLETED**							
No problem	15.5	54.5	22.8	7.1	17.9	15.0	2.0
Slight problems	34.1	25.8	38.1	38	37.3	24.6	18.5
Moderate problems	26.6	13.5	23.6	33.2	26.5	22.2	40.9
Severe problems	16.6	4.1	11.6	13.8	10.1	24.7	31.8
Unable/extreme problems	7.2	2.1	3.9	7.9	8.1	13.5	6.8
The best health (11111)	1.9						
The best health including DG (111111)	1.6						
The best health including BL (111111)	0.7						
The best health including both (1111111)	0.6						
**PROXY-COMPLETED**							
No problem	27.0	54.5	31.1	9.0	22.5	19.4	1.8
Slight problems	33.3	25.2	40.5	39.2	45.0	33.8	24.3
Moderate problems	19.8	13.5	16.2	28.4	19.4	19.8	41.4
Severe problems	11.7	2.3	7.2	13.5	9.9	18.0	28.8
Unable/extreme problems	8.1	4.5	5.0	9.9	3.2	9.0	3.6
The best health (11111)	5.9						
The best health including DG (111111)	3.2						
The best health including BL (111111)	1.4						
The best health including both (1111111)	0.9						

*MO, mobility; SC, self-care; UA, usual activates; PD, pain/discomfort; AD, anxiety/depression; DG, dignity; BL, bleeding*.

### Convergent Validity

**Figure 1:**
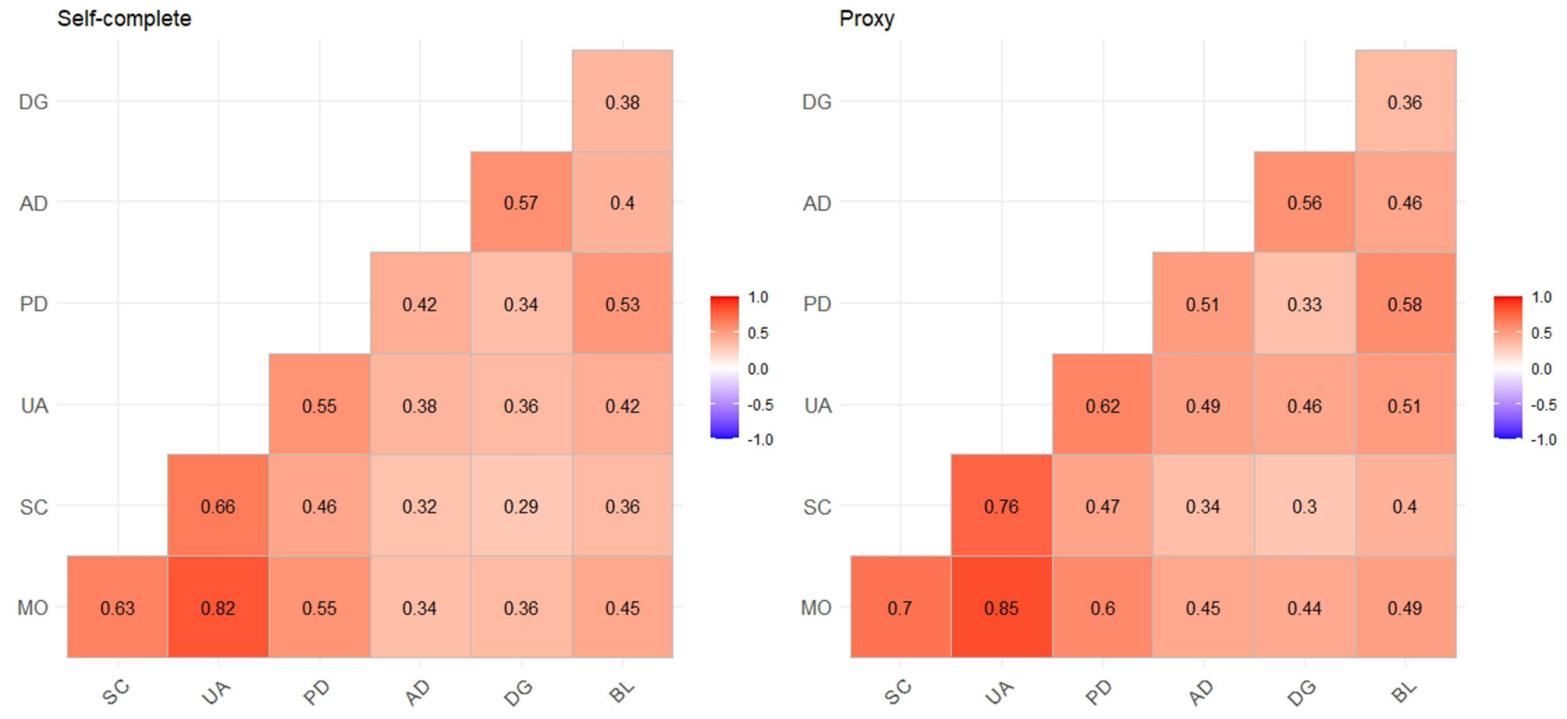
Correlation between EQ-5D items and bolt-on items; MO, mobility; SC, self-care; UA, usual activates; PD, pain/discomfort; AD, anxiety/depression; DG, dignity; BL, bleeding; all the coefficients are statistically significant.

The correlations between the EQ-5D-5L and bolt-on items are shown in Figure 1. For patients who self-completed their questionnaire, the associations of the EQ-5D-5L with the DG and BL items ranged from 0.29 to 0.57 and 0.36 to 0.53, respectively. For patients who had completed their questionnaire by proxy, the associations of the EQ-5D-5L with the DG and BL items were slightly stronger than those of patients who had self-completed their questionnaires. Table 3 demonstrates the convergent validity of the EQ-5D-5L with two bolt-on items. The results confirmed our hypotheses that the EQ-5D-5L with two bolt-on items show a positive relationship with the Haem-A-QoL total score [range: 0.2 to 0.64 (self); 0.27 to 0.64 (proxy)], but a negative relationship with PCS [−0.64 to −0.29 (self); −0.65 to −0.4 (proxy)], and MCS [−0.62 to −0.3 (self); −0.61 to −0.35 (proxy)]. Additionally, the DG item was strongly and significantly correlated with MCS, whereas the BL item showed a stronger correlation with PCS.

**Table 3:** Correlation between EQ-5D-5L, bolt-on items and the Haem-A-QoL and the SF-12.

	**SPEARMAN CORRELATION COEFFICIENT**					
	**Self-completed**			**Proxy-completed**		
	**Haem-A-QoL**	**PCS**	**MCS**	**Haem-A-QoL**	**PCS**	**MCS**
MO	0.38^***^	−0.64^***^	−0.32^***^	0.64^***^	−0.65^***^	−0.49^***^
SC	0.2^**^	−0.47^***^	−0.3^***^	0.47^**^	−0.47^***^	−0.35^***^
UA	0.31^***^	−0.61^***^	−0.35^***^	0.54^**^	−0.62^***^	−0.47^***^
PD	0.42^***^	−0.51^***^	−0.36^***^	0.37^*^	−0.53^***^	−0.41^***^
AD	0.59^***^	−0.29^***^	−0.62^***^	0.27	−0.4^***^	−0.58^***^
DG	0.64^***^	−0.36^***^	−0.61^***^	0.66^***^	−0.41^***^	−0.61^***^
BL	0.49^***^	−0.53^***^	−0.35^***^	0.31^*^	−0.57^***^	−0.39^***^

*MO, mobility; SC, self-care; UA, usual activates; PD, pain/discomfort; AD, anxiety/depression; DG, dignity; BL, bleeding; PCS, physical component summary score; MCS, mental component summary score*.

* *p < 0.05*;

** *p < 0.01*;

*** *p < 0.001*.

### Exploratory Power

Univariable and Multivariable linear regression analysis was used to examine the performance of the models in predicting the VAS scores. The data in Table 4 reveal that the EQ-5D+DG+BL model showed better performance than the other models on the basis of adjusted *R*^2^. Compared with the EQ-5D, adding two bolt-on items increased the explanatory power from 0.294 to 0.318 for patients who self-completed their questionnaires and 0.432 to 0.47 for patients whose questionnaires had been completed by proxy. In other words, the two bolt-on items have significantly increased the instrument's ability to predict EQ-VAS scores. The adjusted *R*^2^ increased by 8.2 and 8.8% for reports completed by the patients or patients' proxy respondents, respectively. In the comparison of the performance of the EQ-5D+DG and the EQ-5D+BL, the former combination explained more variations in VAS scores than the latter, which substantially reduced the adjusted *R*^2^ of 3.2 and 3.7% both patients who self-completed their questionnaire and patients whose questionnaires were completed by proxy, respectively.

**Table 4:** Regression analysis evaluating the impact of adding bolt-on items in predicting EQ-VAS score.

	***R*^**2**^**	**Adjusted *R*^**2**^**	***F*-statistics**	***p*-Value**
**SELF-COMPLETED**				
MO	0.196	0.192	54.11	<0.001
SC	0.144	0.14	37.49	<0.001
UA	0.178	0.175	48.35	<0.001
PD	0.191	0.188	52.65	<0.001
AD	0.154	0.15	40.45	<0.001
DG	0.163	0.159	43.31	<0.001
BL	0.152	0.148	39.9	<0.001
DG+BL	0.23	0.22	33.06	<0.001
MO+SC+UA+PD+AD	0.31	0.294	19.70	<0.001
MO+SC+UA+PD+AD+DG	0.331	0.312	17.97	<0.001
MO+SC+UA+PD+AD+BL	0.321	0.302	17.18	<0.001
MO+SC+UA+PD+AD+DG+BL	0.339	0.318	15.93	<0.001
**PROXY-COMPLETED**				
MO	0.341	0.325	22.32	<0.001
SC	0.228	0.21	12.76	<0.001
UA	0.367	0.352	25.03	<0.001
PD	0.264	0.247	15.52	<0.001
AD	0.267	0.25	15.73	<0.001
DG	0.272	0.255	16.16	<0.001
BL	0.243	0.226	13.88	<0.001
DG+BL	0.37	0.35	15.86	<0.001
MO+SC+UA+PD+AD	0.484	0.432	9.41	<0.001
MO+SC+UA+PD+AD+DG	0.519	0.461	8.87	<0.001
MO+SC+UA+PD+AD+BL	0.504	0.444	8.35	<0.001
MO+SC+UA+PD+AD+DG+BL	0.537	0.470	7.99	<0.001

*MO, mobility; SC, self-care; UA, usual activates; PD, pain/discomfort; AD, anxiety/depression; DG, dignity; BL, bleeding*.

### Known-Group Validity

For both patients who self-completed their questionnaire and patients whose questionnaires were completed by proxy, those who reported hemophilia-related disability, poorer perceived health status, and comorbidity were highly likely to report having extreme problems with both dignity and bleeding. Patients who self-completed their questionnaire showed a higher proportion of reporting extreme problems with dignity and bleeding compared with patients whose questionnaires were completed by proxy. The results confirmed that the two bolt-on items had good discriminatory ability to differentiate patients with different health statuses. The results of the analysis of known-group validity of the HRQoL measurements are shown in Table 5.

**Table 5:** Summary of known-groups validity testing of the two bolt-on items.

	**%**										
	**Overall**	**DG**					**BL**				
		**No problem**	**Slight problem**	**Moderate problem**	**Severe problem**	**Unable/Extreme**	**No problem**	**Slight problem**	**Moderate problem**	**Severe problem**	**Unable/Extreme**
**SELF-COMPLETED**											
**Disability**											
No	37.1	22.8	28.0	21.8	17.3	10.0	3.8	27.0	44.6	20.4	4.2
Yes	62.9	10.6	22.3	22.1	29.2	15.7	1.2	13.7	38.9	37.0	9.2
*p*-value		<0.001					<0.001				
**Severity**											
Minor	27.4	22.0	35.0	20.3	16.3	6.5	5.3	32.5	52.8	8.9	0.4
Moderate	26.5	14.7	22.7	29.8	25.2	7.6	1.3	13.9	48.7	32.8	3.4
Severe	46.1	11.1	19.6	18.8	29.5	21.0	0.5	12.8	29.2	44.9	12.6
*p*-value		<0.001					<0.001				
**Comorbidity**											
No	58.0	18.8	27.8	23.4	20.7	9.2	2.5	21.3	40.3	31.3	4.6
Yes	42.0	9.8	20.2	20.4	30.2	19.4	1.3	14.6	41.6	32.6	9.8
*p*-value		<0.001					0.003				
**PROXY-COMPLETED**											
**Disability**											
No	53.9	27.3	36.4	15.5	12.7	8.2	2.7	33.6	40.0	21.8	1.8
Yes	46.1	13.8	30.9	23.4	24.5	7.4	1.1	12.8	46.8	34.0	5.3
*p*-value		0.03					0.004				
**Severity**											
Minor	25.7	29.8	33.3	15.8	19.3	1.8	3.5	36.8	54.4	5.3	0
Moderate	23.0	15.7	27.5	25.5	23.5	7.8	3.9	27.5	45.1	23.5	0
Severe	51.4	15.8	36.8	19.3	14.9	13.2	0	16.7	33.3	43.0	7.0
*p*-value		0.06					<0.001				
**Comorbidity**											
No	64.9	25.7	38.2	17.4	13.2	5.6	2.8	29.2	43.8	22.2	2.1
Yes	35.1	14.0	25.6	24.4	26.9	15.4	0	15.4	37.2	41.0	6.4
*p*-value		<0.001					0.004				

*DG, dignity; BL, bleeding*.

### Classification Efficiency

The values of H′ for the EQ-5D+DG+BL were higher than those for the EQ-5D-5L, which indicate that the EQ-5D-5L with two bolt-on items generated a larger amount of information. However, the values of *J*′ for the EQ-5D+DG+BL were lower than those for the EQ-5D-5L. These findings suggest that more information is captured by adding two more items to the EQ-5D-5L, but the observed gain in discrimination in both self- and proxy-completed samples is relatively low due to the increase in classification options (Table 6).

**Table 6:** Shannon index (H′) and Shannon Evenness index (J′) of EQ-5D and bolt-on items.

	**H′**	**J′**
**SELF-COMPLETE**		
EQ-5D	8.29	0.71
EQ-5D+DG	8.48	0.66
EQ-5D+BL	8.49	0.66
EQ-5D+DG+BL	8.64	0.62
**PROXY**		
EQ-5D	6.89	0.59
EQ-5D+DG	7.07	0.55
EQ-5D+BL	7.08	0.55
EQ-5D+DG+BL	7.24	0.52

*DG, dignity; BL, bleeding*.

## Discussion

### Principal Findings

This study assessed the impact of adding two condition-specific bolt-on items to the EQ-5D-5L in a sample of Chinese patients with hemophilia and examined the psychometric properties of the measure. Compared with the EQ-5D-5L, a more satisfactory performance of the EQ-5D-5L with two bolt-on items was confirmed in both the self- and proxy-completed samples. We found that, compared with the EQ-5D-5L, adding two items significantly decreased the ceiling effects (68.4 and 84.7% for self- and proxy-completed samples) and significantly increased the discriminative power to differentiate patients with different health statuses. As expected, the DG item strongly correlated with the AD item of the EQ-5D-5L, whereas the BL item strongly correlated with the PD item. Both showed a statistically significant association with the Haem-A-QoL sum score, but the impact on mental and physical HRQoL (SF-12) was different.

Regression models revealed that the EQ-5D-5L with two bolt-on items showed an improvement in the ability to predict VAS scores. The value of the adjusted *R*^2^ increased from 0.294 to 0.318 for patients who self-completed their questionnaires and 0.432 to 0.47 for patients whose questionnaires had been completed by proxy. When comparing the predictive ability of the two bolt-on items, the DG model outperformed the BL model. The adjusted *R*^2^ increased by 6.1% when the DG item (proxy: 6.7%) was added and 2.7% (proxy: 2.8%) when the BL item was added. One possible reason for this finding may be that the DG item was designed to capture the variation of mental HRQoL among patients, which provided extra information that might not have been possible to capture, or fully capture, with the EQ-5D-5L only. The BL item was used to measure the patients' physical HRQoL. Given that four of the five items of the EQ-5D-5L measure physical HRQoL, it is likely that the information gathered with the BL item overlapped with other physical HRQoL items, which strengthened its predictive ability. Moreover, Neufeld et al. found that there are other factors that significantly affect patients' overall HRQoL and day-to-day variability in the non-bleeding state (54).

### Comparison With Previous Studies

Although no studies have targeted hemophilia, adding bolt-on items to the EQ-5D to improve its sensitivity in capturing the variations of HRQoL in different patient groups have been studied before. For example, Gandhi et al. indicated that the bolt-on item of “vision” could increase the responsiveness of the EQ-5D to detect changes in health outcomes among patients undergoing cataract surgery (33). Geraerds et al. also found that adding a cognition item to the EQ-5D could improve its explanatory power and informativity among patients with traumatic brain injury (53). Kangwanrattanakul et al. demonstrated that the bolt-on item of “activities related to bending knees” could improve the sensitivity of the EQ-5D questionnaire and better measure health status among Thai people (31).

In the present study, both the BL and DG items were found to substantially increase the predictive ability of the EQ-5D-5L, and the DG item showed a larger increase than the BL item. This suggests that, besides regaining physical health from the treatment, living with independence, dignity, and self-esteem may be more important among patients with hemophilia (40). Previous studies in the other patient groups showed similar findings. One German study found that low sense of dignity in patients with cancer was significantly associated with psychological distress (55). Baillie and Llott also found that patients feel vulnerable when they experience loss of control, lack of privacy, and insecurity (56). Another qualitative study conducted in the United Kingdom demonstrated that patients with spinal injury must be treated with respect and dignity as long as they need (57). Adding a dignity dimension to the EQ-5D is important in measuring HRQoL, considering that it is a chronic, rather than instantaneous, need of patients. The performance of a dignity item in the EQ-5D in patients with other RDs warrants further investigation.

We noted that the strength of the correlation between the DG and BL items was not very strong, which indicates that patients who feel the least dignity are not those who have the most severe bleeding problems. This is in line with the findings of some cancer studies that have indicated that loss of dignity may be a broader concern among medically ill people and is not limited to patients with very severe conditions or at the end of life (58–60). It is important to note that dignity is a multi-faceted concept; for example, for end-of-life patients, dignity involves quality care to achieve a sense of spiritual peace and well-being (61). However, for patients with moderate or mild conditions, showing them dignity could be more general, for example, sharing decision-making with them, respecting their preferences, protecting their privacy, etc. (62).

Few studies have compared the performance of bolt-on items from the perspective of patients and caregivers. Our findings were consistent with those of previous studies that suggest that patients are likely to overestimate the severity of their health states compared with caregivers or the general public (63). We further found that, compared with caregivers, patients reported suffering more extreme problems on the BL item (47%) than on the DG item (33%). This finding suggests that caregivers, to some extent, are more likely to underestimate the physical HRQoL of hemophilia patients than mental HRQoL. Further, we also find that the EQ-5D items and bolt-on items tended to be statistically insignificant in predicting the Haem-A-QoL scores in the sample of caregiver (Supplementary Table A1). The utility score of the EQ-5D elicited from the perspective of the public or patients is a long-term controversy. The current literature mainly supports that patients tend to assign higher utilities compared with members of the public (64–66). However, few alternative utility algorithms were produced when bolt-on items were included in the EQ-5D (32), which limited our knowledge on the impact of bolt-on items on the values for EQ-5D health states. Further research is needed to develop the utility values of the EQ-5D+BL+DG.

## Strengths and Limitations

This was the first study to develop bolt-on items to improve the sensitivity of the EQ-5D-5L to measure HRQoL in patients with hemophilia worldwide. In addition, this study was one of the very few worldwide, and the first in China, to present and compare the impact and psychometric properties of the bolt-on items to the EQ-5D-5L from the perspectives of patients and proxies. Third, in this study, the development of the bolt-on items to the EQ-5D-5L followed a solid process, which included a literature review, patient interviews, expert discussions, and cognitive debriefing. Finally, we found that dignity is an important issue affecting mental HRQoL and social well-being in patients with RDs. We confirmed that the EQ-5D-5L with dignity showed satisfactory psychometric properties among hemophiliacs, which suggests a further application in the other RD patient groups.

This study also had several limitations that should be addressed. First, data used in this study was collected via an online survey, the patients who were unable to approach Internet were excluded from the survey, which may lead to some selection bias. Additionally, no response rate can be calculated based on the online survey, because only information of successfully submitted respondents were recorded. Neither can we rule out the possibility that some of the participants might have cognitive problems, even though this was one of the eligibility criteria. Second, all the participants in this study were recruited from a national hemophilia patient group, even though it is the largest patient group for hemophilia patients in China, selection bias might have been introduced. Last, in this study, caregivers completed the self-completed rather than proxy version of the EQ-5D, which may weaken the validity of our findings. Finally, all the data were self-reported, which might have caused recall bias, which could limit the generalizability of our findings.

## Conclusions

The EQ-5D-5L with two bolt-on items showed good psychometric properties among Chinese patients with hemophilia in our study. Both items improved the sensitivity and exploratory power of the EQ-5D-5L. The DG item was strongly correlated with hemophilia psychological HRQoL, whereas the BL captured more information related to patients' physical HRQoL. A higher convergent and known-group validity of the EQ-5D-5L was observed in the patients who had self-completed their questionnaire compared with patients who had completed their questionnaire by proxy, suggesting that the additional value of condition-specific items to the EQ-5D-5L was more relevant in the self-completed sample. This study was designed as the first stage in an assessment of the influence of bolt-on items on health states covering a range of RDs in China. The second stage of the research would be to undertake a valuation study to facilitate an estimate of the value algorithm for the EQ-5D-5L with bolt-on items. Further, the performance of the dignity will be investigated in patients with the other RDs.

## Data Availability Statement

The raw data supporting the conclusions of this article will be made available by the authors, without undue reservation.

## Ethics Statement

The studies involving human participants were reviewed and approved by the institutional review board ethics committee of the Chinese University of Hong Kong and Peking Union Medical College Hospital approved the study protocol and informed consent (Ref no.: SBRE-18-268 and SK814). The patients/participants provided their written informed consent to participate in this study.

## Author Contributions

RX: material preparation, data collection, draft preparation, data analysis, and manuscript revision. DD: study conception and design, material preparation, data collection, data analysis, manuscript revision, and supervision. NL: data analysis and manuscript revision. RY and JL: manuscript revision. SZ: study conception and design, manuscript revision, supervision, and funding. All authors contributed to the article and approved the submitted version.

## Conflict of Interest

The authors declare that the research was conducted in the absence of any commercial or financial relationships that could be construed as a potential conflict of interest.

## Publisher's Note

All claims expressed in this article are solely those of the authors and do not necessarily represent those of their affiliated organizations, or those of the publisher, the editors and the reviewers. Any product that may be evaluated in this article, or claim that may be made by its manufacturer, is not guaranteed or endorsed by the publisher.

## References

[1] Srivastava A Brewer AK Mauser-Bunschoten EP Key NS Kitchen S Llinas A. Guidelines for the management of hemophilia. Haemophilia. (2013) 19:e1–47. 10.1111/j.1365-2516.2012.02909.x 22776238

[2] Stoffman J Andersson NG Branchford B Batt K D'Oiron R Escuriola Ettingshausen C. Common themes and challenges in hemophilia care: a multinational perspective. Hematology. (2018) 24:39–48. 10.1080/10245332.2018.1505225 30073913

[3] Trindade GC Viggiano LG de L Brant ER Lopes CAO Faria ML Ribeiro PHNS. Evaluation of quality of life in hemophilia patients using the WHOQOL-bref and Haemo-A-Qol questionnaires. Hematol Transfus cell Ther. (2019) 41:335–41. 10.1016/j.htct.2019.03.010 31409581 PMC6978543

[4] Hemophilia Prognosis and Life Expectancy. Available online at: https://hemophilianewstoday.com/hemophilia-prognosis-life-expectancy/ (accessed June 15, 2021).

[5] Mayo Clinics. Hemophilia - Symptoms and Causes. Available online at: https://www.mayoclinic.org/diseases-conditions/hemophilia/symptoms-causes/syc-20373327 (accessed March 1, 2021).

[6] O'Hara J Walsh S Camp C Mazza G Carroll L Hoxer C. The impact of severe haemophilia and the presence of target joints on health-related quality-of-life. Health Qual Life Outcomes. (2018). 16:84. 10.1186/s12955-018-0908-9 29720192 PMC5932770

[7] Mulder K Llinas A. The target joint. Haemophilia. (2004) 10:152–6. 10.1111/j.1365-2516.2004.00976.x 15479389

[8] McLaughlin JM Munn JE Anderson TL Lambing A Tortella B Witkop ML. Predictors of quality of life among adolescents and young adults with a bleeding disorder. Health Qual Life Outcomes. (2017) 15:67–9. 10.1186/s12955-017-0643-7 28388906 PMC5383972

[9] Scalone L Mantovani LG Mannucci PM Gringeri A; COCIS Study Investigators. Quality of life is associated to the orthopaedic status in haemophilic patients with inhibitors. Haemophilia. (2006) 12:154–62. 10.1111/j.1365-2516.2006.01204.x 16476090

[10] Elander J Morris J Robinson G. Pain coping and acceptance as longitudinal predictors of health-related quality of life among people with haemophilia-related joint pain. Eur J Pain. (2013) 17:929–38. 10.1002/j.1532-2149.2012.00258.x 23242704

[11] Saba HI Tran DQ Jr. Challenges and successes in the treatment of hemophilia: the story of a patient with severe hemophilia A and high-titer inhibitors. J Blood Med. (2012) 3:17–23. 10.2147/JBM.S30479 22715320 PMC3370834

[12] von der Lippe C Diesen PS Feragen KB. Living with a rare disorder: a systematic review of the qualitative literature. Mol Genet genomic Med. (2017). 5:758–73. 10.1002/mgg3.315 29178638 PMC5702559

[13] Weill A. Haemophilia treatment for all and the role of tolerance, difference and education. Haemophilia. (2017) 23:341–3. 10.1111/hae.13213 28520202

[14] Barclay L. In sickness and in dignity: a philosophical account of the meaning of dignity in health care. Int J Nurs Stud. (2016) 61:136–41. 10.1016/j.ijnurstu.2016.06.010 27351830

[15] Ganzini L Goy ER Dobscha SK. Why oregon patients request assisted death: family members' views. J Gen Intern Med. (2008) 23:1296. 10.1007/s11606-008-0542-z 18080719 PMC2265314

[16] Chochinov HM Kristjanson LJ Hack TF Hassard T McClement S Harlos M. Burden to others and the terminally ill. J Pain Symptom Manage. (2007) 34:463–71. 10.1016/j.jpainsymman.2006.12.012 17616329

[17] Chochinov HM. Dignity-conserving care–a new model for palliative care: helping the patient feel valued. JAMA. (2002) 287:2253–60. 10.1001/jama.287.17.2253 11980525

[18] Albers Gwenda Ms Pasman H. Roeline W. P Deliens Luc P. Does Health status affect perceptions of factors influencing dignity at the end of life? J Pain Symptom Manage. (2013). 45:1030–8. 10.1016/j.jpainsymman.2012.06.012 23141880

[19] Ramos-Goñi JM Oppe M Slaap B Busschbach JJV Stolk E. Quality control process for EQ-5D-5L valuation studies. Value Heal. (2017) 20:466–73. 10.1016/j.jval.2016.10.012 28292492

[20] Sullivan PW Ghushchyan VH. EQ-5D Scores for diabetes-related comorbidities. Value Heal. (2016) 19:1002–8. 10.1016/j.jval.2016.05.018 27987626

[21] Rencz F Gulácsi L Drummond M Golicki D Prevolnik Rupel V Simon J. EQ-5D in Central and Eastern Europe: 2000–2015. Qual Life Res. (2016) 25:2693–710. 10.1007/s11136-016-1375-6 27472992

[22] Rowen D Azzabi Zouraq I Chevrou-Severac H van Hout B. International regulations and recommendations for utility data for health technology assessment. Pharmacoeconomics. (2017) 35:11–9. 10.1007/s40273-017-0544-y 29052162

[23] Soucie JM Grosse SD Siddiqi AE Byams V Thierry J Zack MM. The effects of joint disease, inhibitors and other complications on health-related quality of life among males with severe haemophilia A in the United States. Haemophilia. (2017). 23:e287–93. 10.1111/hae.13275 28574229 PMC5533283

[24] Carroll L Benson G Lambert J Benmedjahed K Zak M Lee XY. Real-world utilities and health-related quality-of-life data in hemophilia patients in France and the United Kingdom. Patient Prefer Adherence. (2019) 13:941–57. 10.2147/PPA.S202773 31354248 PMC6585419

[25] Abdin E Subramaniam M Vaingankar JA Luo N Chong SA. Measuring health-related quality of life among adults in Singapore: population norms for the EQ-5D. Qual Life Res. (2013) 22:2983–91. 10.1007/s11136-013-0405-x 23549857

[26] Brazier JE Rowen D Lloyd A Karimi M. Future directions in valuing benefits for estimating QALYs: is time up for the EQ-5D? Value Heal. (2019) 22:62–8. 10.1016/j.jval.2018.12.001 30661635

[27] Brazier J. Measuring and Valuing Health Benefits for Economic Evaluation. 2nd ed. Oxford : Oxford University Press. (2007).

[28] Tosh J Brazier J Evans P Longworth L. A review of generic preference-based measures of health-related quality of life in visual disorders. Value Heal. (2012) 15:118–27. 10.1016/j.jval.2011.08.002 22264979 PMC3268858

[29] Brazier J Yang Y Tsuchiya A Rowen DL. A review of studies mapping (or cross walking) non-preference based measures of health to generic preference-based measures. Heal Econ Prev Care. (2010) 11:215–25. 10.1007/s10198-009-0168-z 19585162

[30] Brazier J Connell J Papaioannou D Mukuria C Mulhern B Peasgood T. A systematic review, psychometric analysis and qualitative assessment of generic preference-based measures of health in mental health populations and the estimation of mapping functions from widely used specific measures. Health Technol Assess. (2014). 18:vii–viii, xiii–xxv, 1–188. 10.3310/hta18340 24857402 PMC4781324

[31] Kangwanrattanakul K Gross CR Sunantiwat M Thavorncharoensap M. Exploration of a cultural-adaptation of the EQ-5D for Thai population: a “bolt-on” experiment. Qual Life Res. (2019) 28:1207–15. 10.1007/s11136-018-2072-4 30519906

[32] Yang Y Rowen D Brazier J Tsuchiya A Young T Longworth L. An exploratory study to test the impact on three “bolt-on” items to the EQ-5D. Value Heal. (2015) 18:52–60. 10.1016/j.jval.2014.09.004 25595234 PMC4309886

[33] Gandhi M Ang M Teo K Wong CW Wei YC Tan RL. A vision ‘bolt-on’ increases the responsiveness of EQ-5D: preliminary evidence from a study of cataract surgery. Eur J Heal Econ. (2020) 21:501–11. 10.1007/s10198-019-01156-w 31902023

[34] Brazier J Tsuchiya A. Preference-based condition-specific measures of health: what happens to cross programme comparability? Health Econ. (2010) 19:125–9. 10.1002/hec.1580 20049843

[35] Finch AP Brazier JE Mukuria C. Selecting bolt-on dimensions for the EQ-5D: examining their contribution to health-related quality of life. Value Heal. (2019) 22:50–61. 10.1016/j.jval.2018.07.001 30661634

[36] Pearson I Rothwell B Olaye A Knight C. Economic modeling considerations for rare diseases. Value Heal. (2018) 21:515–24. 10.1016/j.jval.2018.02.008 29753347

[37] Czech M Baran-Kooiker A Atikeler K Demirtshyan M Gaitova K Holownia-Voloskova M. A review of rare disease policies and orphan drug reimbursement systems in 12 Eurasian countries. Front Public Heal. (2020) 7:416. 10.3389/fpubh.2019.00416 32117845 PMC6997877

[38] Herdman M Gudex C Lloyd A Janssen M Kind P Parkin D. Development and preliminary testing of the new five-level version of EQ-5D (EQ-5D-5L). Qual Life Res. (2011) 20:1727–36. 10.1007/s11136-011-9903-x 21479777 PMC3220807

[39] Beach MC Sugarman J Johnson RL Arbelaez JJ Duggan PS Cooper LA. Do patients treated with dignity report higher satisfaction, adherence, and receipt of preventive care? Ann Fam Med. (2005) 3:331–8. 10.1370/afm.328 16046566 PMC1466898

[40] Jacelon CS Connelly TW Brown R Proulx K Vo T. A concept analysis of dignity for older adults. J Adv Nurs. (2004) 48:76–83. 10.1111/j.1365-2648.2004.03170.x 15347413

[41] Baillie L Gallagher A Wainwright P. Defending Dignity – Challenges and Opportunities for Nursing. Royal College of Nursing (2008).

[42] Dixon S Palfreyman S Shackley P Brazier J. What is dignity? A literature review and conceptual mapping (2011). Available online at: http://eprints.whiterose.ac.uk/43280/ (accessed March 1, 2021).

[43] Centers for Disease Control and Prevention. What is Hemophilia. Centers for Disease Control and Prevention. (2020). Available online at: https://www.cdc.gov/ncbddd/hemophilia/facts.html (accessed March 1, 2021).

[44] Mackensen S Catalani O Asikanius E Paz-Priel I Lehle M Trask P. Determining meaningful health-related quality-of-life improvement in persons with haemophilia A using the Haemophilia Quality of Life Questionnaire for Adults (Haem-A-QoL). Haemophilia. (2020) 26:1019–30. 10.1111/hae.14184 33084166

[45] Xu RH Dong D Luo N Wong EL Wu Y Yu S. Evaluating the psychometric properties of the EQ-5D-5L and SF-6D among patients with haemophilia. Eur J Heal Econ. (2021) 22:547–57. 10.1007/s10198-021-01273-5 33761029

[46] Mackensen S Eldar-Lissai A Auguste P Krishnan S von Maltzahn R Yu R. Measurement properties of the Haem-A-QoL in haemophilia clinical trials. Haemophilia. (2017) 23:383–91. 10.1111/hae.13140 28026074

[47] Mackensen S V Gringeri A. Development and pilot testing of a disease-specific quality of life questionnaire for adult patients with haemophilia (Haem-A-QoL). Blood. (2004). 104:608A–9A. 10.1182/blood.V104.11.2214.2214

[48] Von Mackensen S Czepa D Herbsleb M Hilberg T. Development and validation of a new questionnaire for the assessment of subjective physical performance in adult patients with haemophilia – the HEP-Test-Q. Haemophilia. (2010) 16:170–8. 10.1111/j.1365-2516.2009.02112.x 19845778

[49] Shah RM Banahan BF III Holmes ER Patel AS Barnard M Khanna R. An evaluation of the psychometric properties of the sf-12v2 health survey among adults with hemophilia. Health Qual Life Outcomes. (2018) 16:229. 10.1186/s12955-018-1059-8 30545375 PMC6293608

[50] Poon J-L Doctor JN Nichol MB. Longitudinal changes in health-related quality of life for chronic diseases: an example in hemophilia A. J Gen Intern Med. (2014) 29(Suppl 3):S760–6. 10.1007/s11606-014-2893-y 25029975 PMC4124124

[51] Ware JE Kosinski M Keller SD. A 12-item short-form health survey: construction of scales and preliminary tests of reliability and validity. Med Care. (1996) 34:220–33. 10.1097/00005650-199603000-00003 8628042

[52] DeVellis RF. Scale Development : Theory and Applications. 4th ed. Los Angeles, CA: SAGE. (2017).

[53] Geraerds A Bonsel G Janssen M de Jongh MA Spronk I Polinder S. The added value of the EQ-5D with a cognition dimension in injury patients with and without traumatic brain injury. Qual Life Res. (2019) 28:1931–9. 10.1007/s11136-019-02144-6 30820809 PMC6571097

[54] Neufeld EJ Recht M Sabio H Saxena K Solem CT Pickard AS. Effect of acute bleeding on daily quality of life assessments in patients with congenital hemophilia with inhibitors and their families: observations from the dosing observational study in hemophilia. Value Heal. (2012) 15:916–25. 10.1016/j.jval.2012.05.005 22999142

[55] Oechsle K Wais MC Vehling S Bokemeyer C Mehnert A. Relationship between symptom burden, distress, and sense of dignity in terminally ill cancer patients. J Pain Symptom Manage. (2014) 48:313–21. 10.1016/j.jpainsymman.2013.10.022 24766742

[56] Baillie L Ilott L. Promoting the dignity of patients in perioperative practice. J Perioper Pract. (2010) 20:278–82. 10.1177/175045891002000802 20860187

[57] Davis RE Vincent C Henley A McGregor A. Exploring the care experience of patients undergoing spinal surgery: a qualitative study. J Eval Clin Pract. (2013) 19:132–8. 10.1111/j.1365-2753.2011.01783.x 22029534

[58] Solomon BK Wilson KG Henderson PR Poulin PA Kowal J McKim DA. Loss of dignity in severe chronic obstructive pulmonary disease. J Pain Symptom Manage. (2015) 51:529–37. 10.1016/j.jpainsymman.2015.11.007 26620235

[59] Koppelman ER. Dementia and dignity: towards a new method of surrogate decision making. J Med Philos. (2002) 27:65–85. 10.1076/jmep.27.1.65.2971 11961687

[60] Horton R. Rediscovering human dignity. Lancet. (2004) 364:1081–5. 10.1016/S0140-6736(04)17065-7 15380969

[61] Chochinov HM Hack T Hassard T Kristjanson LJ McClement S Harlos M. Dignity therapy: a novel psychotherapeutic intervention for patients near the end of life. J Clin Oncol. (2005) 23:5520–5. 10.1200/JCO.2005.08.391 16110012

[62] Bagheri H Yaghmaei F Ashktorab T Zayeri F. Patient dignity and its related factors in heart failure patients. Nurs Ethics. (2012) 19:316–27. 10.1177/0969733011425970 22354811

[63] Peeters Y Vliet Vlieland TPM Stiggelbout AM. Focusing illusion, adaptation and EQ-5D health state descriptions: the difference between patients and public. Heal Expect. (2012) 15:367–78. 10.1111/j.1369-7625.2011.00667.x 21366809 PMC5060627

[64] Bremner KE Chong CA Tomlinson G Alibhai SM Krahn MD. A review and meta-analysis of prostate cancer utilities. Med Decis Mak an Int J Soc Med Decis Mak. (2007) 27:288–98. 10.1177/0272989X07300604 17502448

[65] Peeters Y Stiggelbout AM. Health state valuations of patients and the general public analytically compared: a meta-analytical comparison of patient and population health state utilities. Value Heal. (2010) 13:306–9. 10.1111/j.1524-4733.2009.00610.x 19744288

[66] De Wit GA Busschbach JJ V De Charro FT. Sensitivity and perspective in the valuation of health status: whose values count? Health Econ. (2000). 9:109–26. 10.1002/(SICI)1099-1050(200003)9:2&lt;109::AID-HEC503&gt;3.0.CO;2-L 10721013

