# Supplementary material for: Investigating the Added Value of the EQ-5D-5L With Two Bolt-On Items in Patients With Hemophilia
Source: Front Med (Lausanne). 2021 Aug 5;8:707998. doi: 10.3389/fmed.2021.707998 (PMC8374886; doi:10.3389/fmed.2021.707998)
Supplement: Supplementary file 1 [file Table_1.DOCX]

Table A1 regression analysis evaluating the impact of adding bolt-on items in predicting Haem-A-QoL score

|  | **R^2^** | **Adjusted R^2^** | **F statistics** | **p-value** |
| --- | --- | --- | --- | --- |
| **Self-completed** |  |  |  |  |
| MO | 0.19 | 0.18 | 11.16 | <0.001 |
| SC | 0.09 | 0.07 | 4.55 | 0.001 |
| UA | 0.15 | 0.13 | 8.03 | <0.001 |
| PD | 0.18 | 0.17 | 10.49 | <0.001 |
| AD | 0.36 | 0.35 | 25.98 | <0.001 |
| DG | 0.41 | 0.4 | 32.32 | <0.001 |
| BL | 0.25 | 0.23 | 15.15 | <0.001 |
| DG+BL | 0.51 | 0.49 | 23.22 | <0.001 |
| MO+SC+UA+PD+AD | 0.48 | 0.42 | 8.24 | <0.001 |
| MO+SC+UA+PD+AD+DG | 0.57 | 0.51 | 9.52 | <0.001 |
| MO+SC+UA+PD+AD+BL | 0.52 | 0.46 | 7.89 | <0.001 |
| MO+SC+UA+PD+AD+DG+BL | 0.6 | 0.53 | 8.97 | <0.001 |
| **Proxy** |  |  |  |  |
| MO | 0.45 | 0.36 | 5.29 | 0.003 |
| SC | 0.28 | 0.21 | 3.61 | 0.03 |
| UA | 0.45 | 0.37 | 5.34 | 0.003 |
| PD | 0.17 | 0.04 | 1.33 | 0.3 |
| AD | 0.16 | 0.03 | 1.26 | 0.31 |
| DG | 0.45 | 0.36 | 5.25 | 0.003 |
| BL | 0.12 | 0.02 | 1.19 | 0.33 |
| DG+BL | 0.5 | 0.34 | 3.26 | 0.01 |
| MO+SC+UA+PD+AD |  |  |  |  |
| MO+SC+UA+PD+AD+DG | 0.76 | 0.21 | 1.38 | 0.32 |
| MO+SC+UA+PD+AD+BL | 0.78 | 0.35 | 1.8 | 0.17 |
| MO+SC+UA+PD+AD+DG+BL | 0.85 | 0.27 | 1.45 | 0.33 |
